# Supplementary material for: DHX36 prevents the accumulation of translationally inactive mRNAs with G4-structures in untranslated regions
Source: Nat Commun. 2019 Jun 3;10:2421. doi: 10.1038/s41467-019-10432-5 (PMC6547686; doi:10.1038/s41467-019-10432-5)
Supplement: Supplementary file 1 — Supplementary Information [file 41467_2019_10432_MOESM1_ESM.pdf]

Supplementary Information

DHX36 prevents the accumulation of translationally inactive mRNAs with G4-structures in untranslated regions

Sauer, Juranek, Marks, De Magis et al.

Supplementary Figures (1-9) and Supplementary Table 1

Supplementary Figures

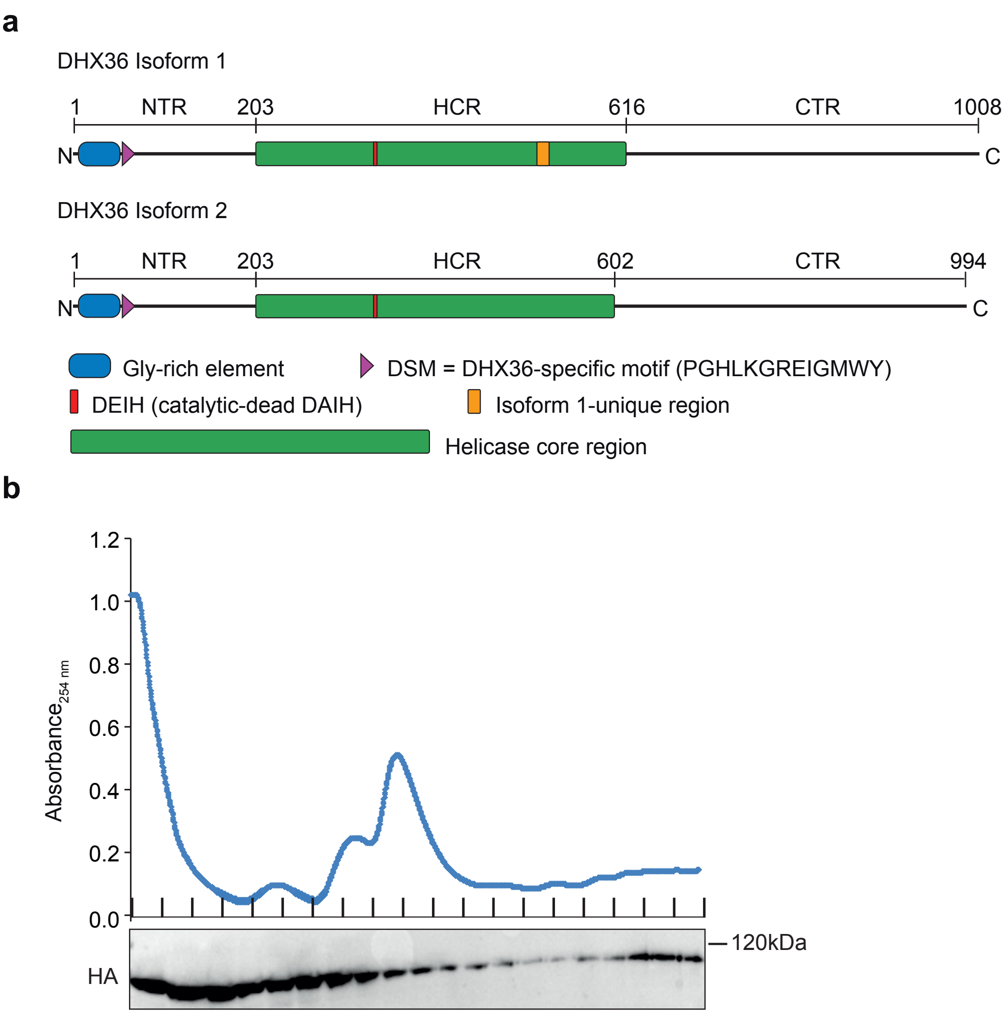

Supplementary Figure 1. Schematic of DHX36 isoforms and DHX36 overexpression polysome gradient. a, Schematic representation of DHX36 isoforms 1 and 2. The RNA binding Gly-rich element (blue) and DHX36-specific motif (DSM) (purple) are indicated. 14 additional aa in the helicase core region (green) of isoform 1 are marked (orange). The conserved Walker-B box is indicated in red (wildtype sequence: DEIH, catalytically inactive mutant sequence: DAIH). **b,** UV absorbances at 254 nm DHX36 overexpression HEK293 cell extracts separated by sucrose gradient centrifugation are shown. Western blot probed for FH-DHX36 is shown below. Source data are provided as a Source Data file.

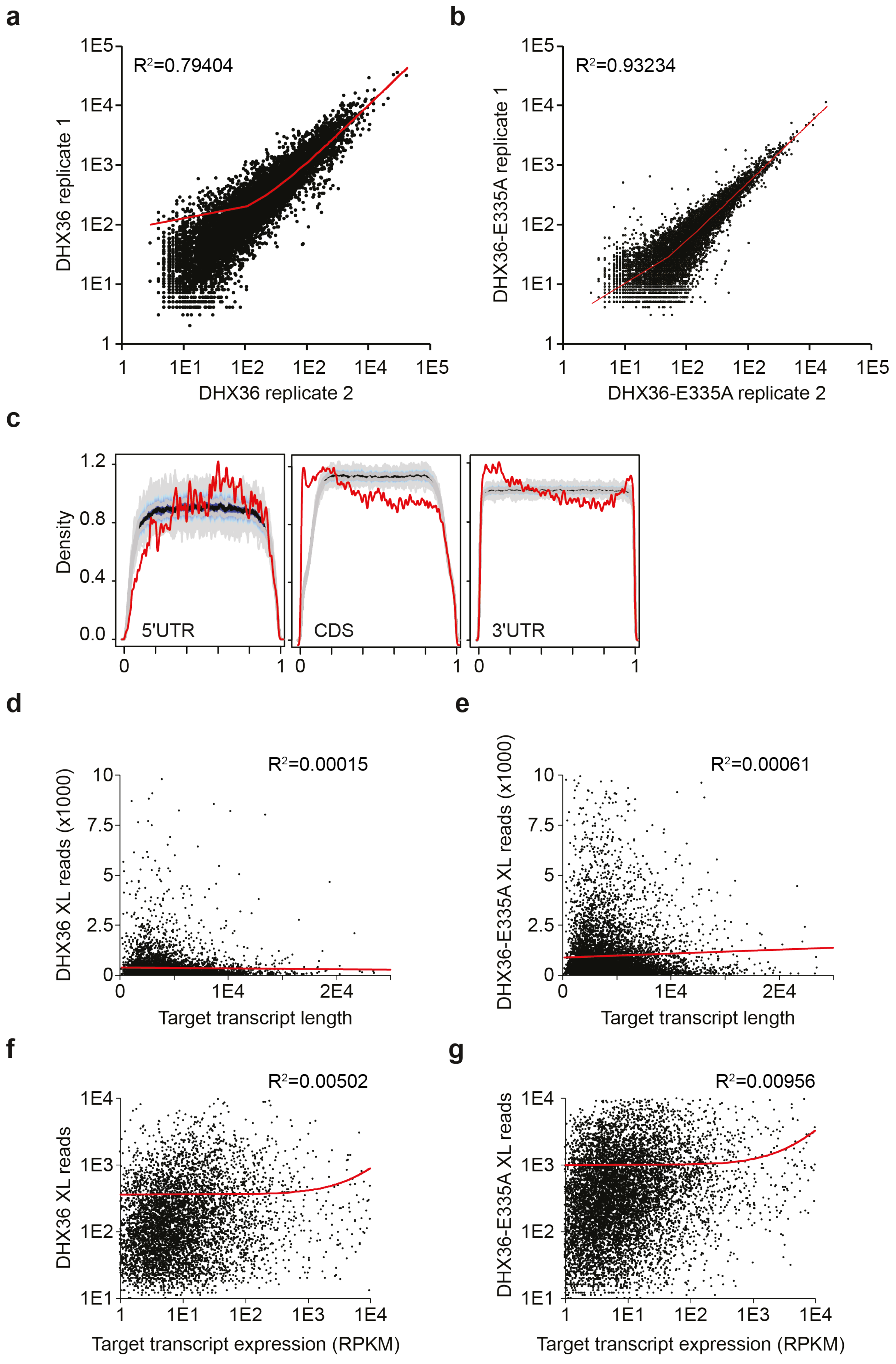
a
R²=0.79404
DHX36 replicate 1
DHX36 replicate 2
1E5
1E4
1E3
1E2
1E1
1
1E1
1E2
1E2
1E4
1E5
b
R²=0.93234
DHX36-E335A replicate 1
DHX36-E335A replicate 2
c
Density
1.2
0.8
0.4
0.0
5'UTR
CDS
3'UTR
0
1
d
R²=0.00015
DHX36 XL reads (x1000)
10
7.5
5
2.5
0
1E4
2E4
Target transcript length
e
R²=0.00061
DHX36-E335A XL reads (x1000)
Target transcript length
f
R²=0.00502
DHX36 XL reads
1E3
Target transcript expression (RPKM)
g
R²=0.00956
DHX36-E335A XL reads
Target transcript expression (RPKM)

Supplementary Figure 2. Replicates of FH-DHX36 and FH-DHX36-E335A PAR-CLIPs and binding determinants. a - b, The correlation of crosslinked reads from the biological replicates of FH-DHX36 **(a)** or FH-DHX36-E335A **(b)** PAR-CLIP experiments is shown. **c,** DHX36 binds preferentially close to the start and the stop codon based on a metagene analysis of the distribution of DHX36 binding clusters on mRNAs subdivided into 5' UTR, CDS, and 3' UTR (red lines). The distribution of 1,000 mismatched randomized controls is shown in gray lines. **d – g,** DHX36 binding on target mRNA is not determined by target transcript length or expression. **d - e,** Correlation between crosslinked reads from FH-DHX36 PAR-CLIP **(d)** or FH-DHX36-E335A PAR-CLIP **(e)** and target transcript length. Correlation coefficients (R^2) are indicated. **f - g,** DHX36 binding on target mRNA is also not determined by target transcript expression. Correlation of DHX36 binding with target transcript expression from FH-DHX36 PAR-CLIP **(f)** or FH-DHX36-E335A PAR-CLIP **(g)**.

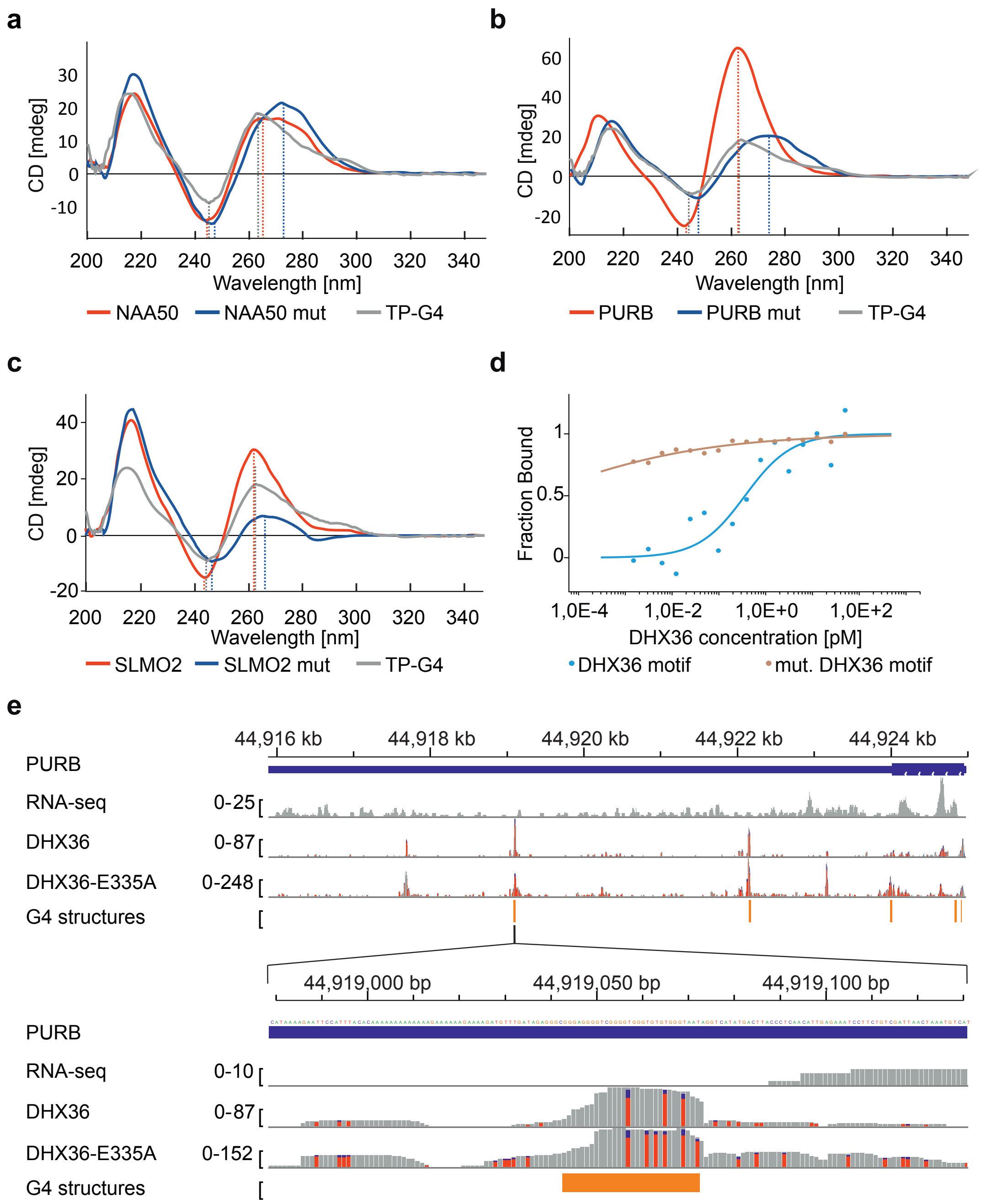

Supplementary Figure 3. DHX36 binds at G-rich sites which can form a parallel G-quadruplex *in vitro*. a - c, Circular dichroism measurements of oligonucleotides representing native DHX36 RRE of the NAA50 **(a)**, PURB **(b)**, and SLMO2 **(c)** mRNAs after performing the G4-folding protocol. Positive control TP-G4) is indicated in gray, wildtype RREs in red, and mutated RREs in blue. Lines represent mean of ten subsequent measurements. Sequences of oligonucleotides are listed Suppl. Table S7. **d,** Microscale thermophoresis analysis shows binding of DHX36 to the DHX36-binding motif (Cy5-AAAAAGGAGGAGGAGGAGG) but not to the mutated motif (Cy5-AAAAAGCAGCAGGAGCAGCA). **e,** Top panel: Screenshots of the FH-DHX36 and FH-DHX36-E335A PAR-CLIP binding sites for the

representative target gene PURB. The gene structure is shown, as well as coverage from a HEK293 RNA-seq experiment. The bottom two tracks show the alignment of sequence reads with characteristic T-to-C mutations from a FH-DHX36 and FH-DHX36-E335A PAR-CLIP experiment. Bottom panel: Close-up of the indicated region in the 3' UTR of PURB. Source data are provided as a Source Data file.

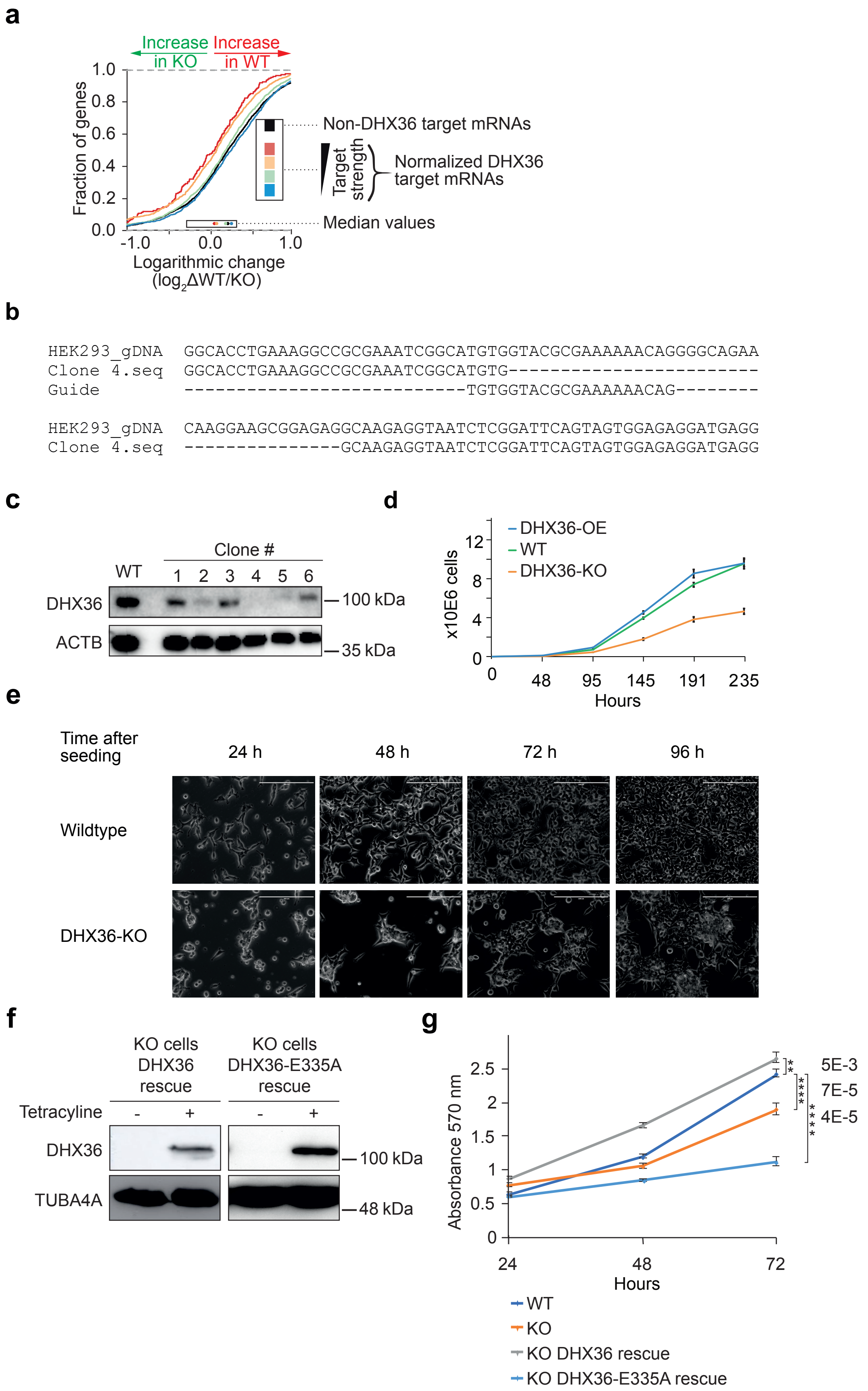

Supplementary Figure 4. Characterization of the DHX36-KO cell line. a, Schematic explanation of cumulative distribution functions (CDFs). mRNAs are grouped in “DHX36 non-targets” (black line) and

according to the binding strength sorted by colors (red, yellow, green, and blue) represented by normalized crosslinked reads per million (NXPM) or the number of binding sites (clusters, in the Supplements). Conditions of binning, number (n) and significances are indicated. Fractions of genes are blotted against the logarithmic change of wildtype over DHX36-KO. Shifting of a colored line to the left compared to the non-targets (black) means higher amounts in wildtype compared to the DHX36-KO and shifting to the right means higher amounts in DHX36-KO compared to wildtype, respectively. Colored dots on the x-axis represent median values. **b,** Sequence of genomic DNA of DHX36 knockout clone indicates the disruption of the gene. Sequence of guide RNA (gRNA) is indicated. **c,** Western blot screening after Cas9-mediated gene editing shows that DHX36 was knocked out in clones 4 and 5. ACTB serves as loading control. **d,** DHX36-KO cells have a growth defect compared to wildtype. Error bars represent standard deviations of three independent experiments. **e,** Changes in morphology of DHX36-KO cells compared to parental wildtype HEK293 cells. KO cells are not able to equally spread over the culture plate surface. Images were taken at indicate timepoints after seeding. Bar represents 200 μm. **f,** Generation of DHX36-KO cells stably expressing FH-DHX36 or FH-DHX36-E335A, respectively, upon tetracycline induction. **g,** MTT-proliferation assay depicted by blotting absorbance at 570 nm (maximum of the formazan product) over time (range of 72 h). HEK293 WT cells have higher proliferation rates compared to DHX36-KO cell. KO cells overexpressing DHX36-E335A have even lower proliferation rates as paternal DHX36-KO cells. Proliferation rates of KO cells overexpressing DHX36 exceed wildtype rates (n=3). Error bars represent standard deviations of four independent experiments. Source data are provided as a Source Data file.

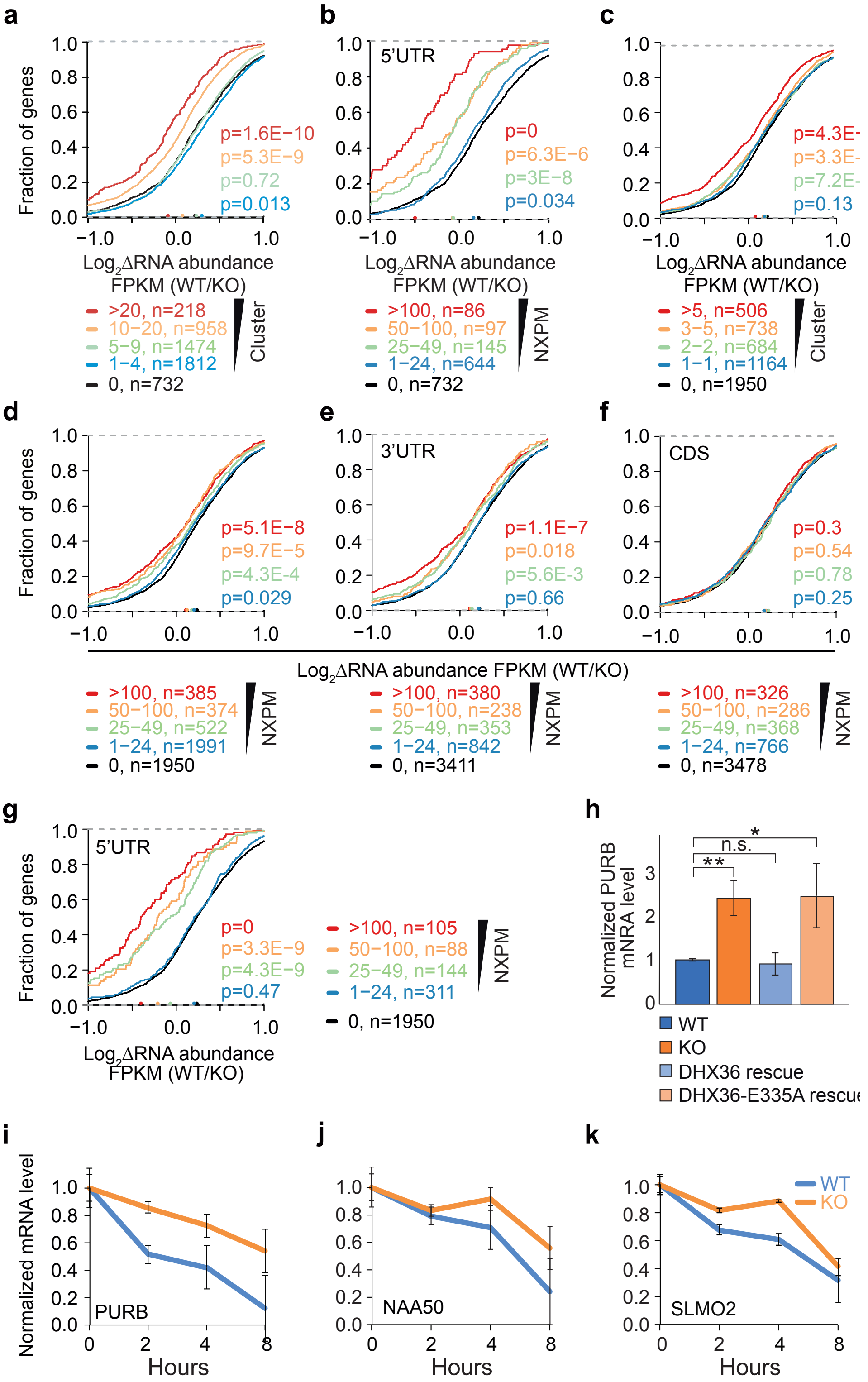

Supplementary Figure 5. Detailed integration of PAR-CLIP with RNA-seq data. a, Same as in **Fig. 4a**, except mRNAs were binned based on the number of binding clusters. **b,** Same as in **Fig. 4a**, except mRNAs were binned based on the number of NXPM in the 5' UTR. **c,** Same as in **a**, except FH-DHX36 wildtype PAR-CLIP data were used. **d,** Same as in **Fig. 4a**, except FH-DHX36 wildtype PAR-CLIP data were used. **e,** Same as in **Fig. 4c**, except FH-DHX36 wildtype PAR-CLIP data were used. **f,** Same as

in **Fig. 4b**, except FH-DHX36 wildtype PAR-CLIP data were used. **g,** Same as in **b**, except FH-DHX36 wildtype PAR-CLIP data were used. **h,** Same as in **Fig. 4e**, except analyzing PURB mRNA levels. **I - k,** Same as in **Fig. 4g** except DHX36 binding target mRNAs PURB (**i**), NAA50 (**j**), and SLMO2 (**k**) were analyzed. Error bars represent standard deviations of three independent experiments. Source data are provided as a Source Data file.

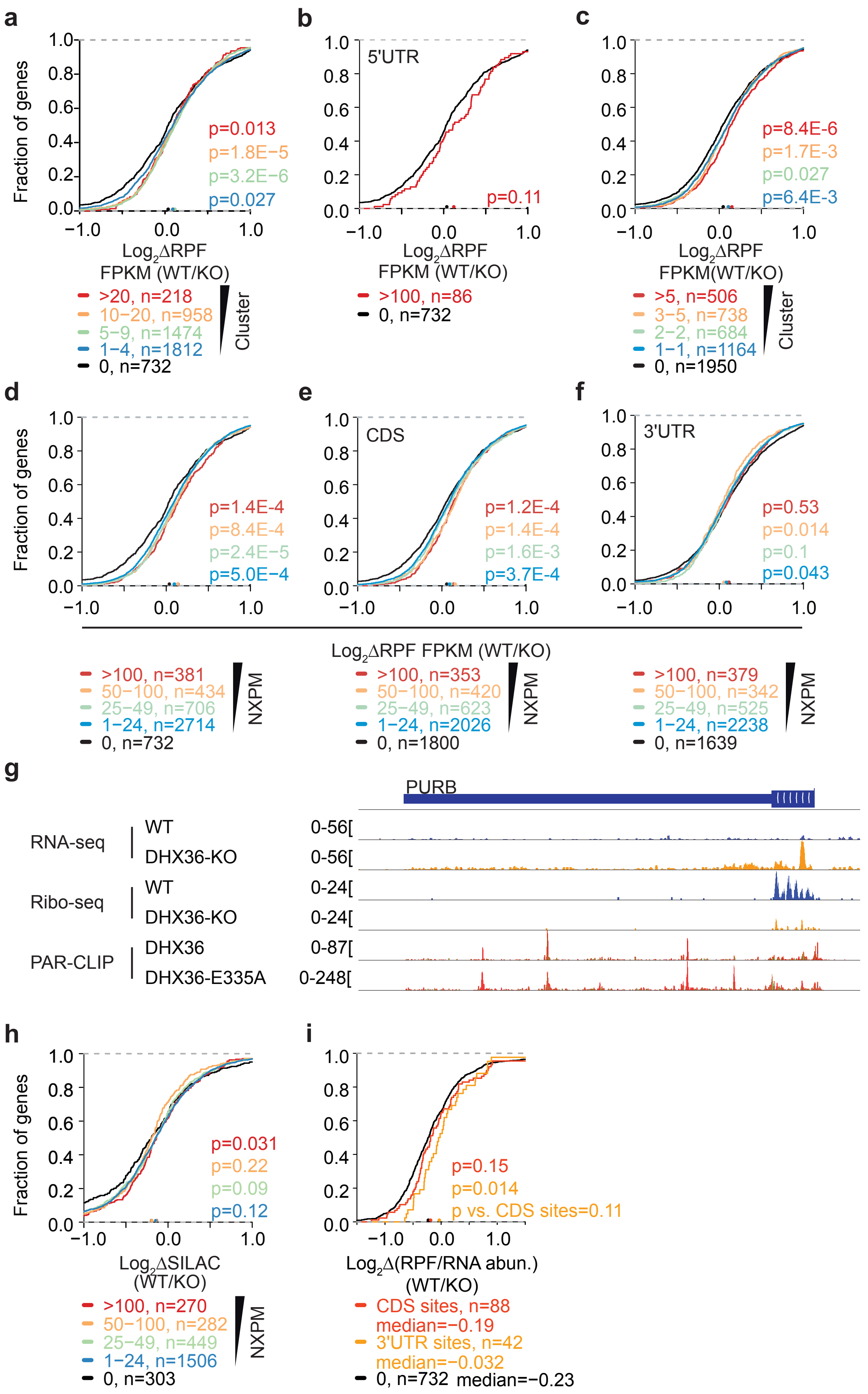
a
Fraction of genes
Log2ΔRPF FPKM (WT/KO)
p=0.013
p=1.8E−5
p=3.2E−6
p=0.027
>20, n=218
10−20, n=958
5−9, n=1474
1−4, n=1812
0, n=732
Cluster
b
5'UTR
p=0.11
Log2ΔRPF FPKM (WT/KO)
>100, n=86
0, n=732
c
p=8.4E-6
p=1.7E-3
p=0.027
p=6.4E-3
Log2ΔRPF FPKM(WT/KO)
>5, n=506
3−5, n=738
2−2, n=684
1−1, n=1164
0, n=1950
Cluster
d
Fraction of genes
p=1.4E-4
p=8.4E-4
p=2.4E-5
p=5.0E-4
e
CDS
p=1.2E-4
p=1.4E-4
p=1.6E-3
p=3.7E-4
f
3'UTR
p=0.53
p=0.014
p=0.1
p=0.043
Log2ΔRPF FPKM (WT/KO)
>100, n=381
50−100, n=434
25−49, n=706
1−24, n=2714
0, n=732
NXPM
>100, n=353
50−100, n=420
25−49, n=623
1−24, n=2026
0, n=1800
NXPM
>100, n=379
50−100, n=342
25−49, n=525
1−24, n=2238
0, n=1639
NXPM
g
PURB
RNA-seq
WT
DHX36-KO
Ribo-seq
WT
DHX36-KO
PAR-CLIP
DHX36
DHX36-E335A
0-56[
0-56[
0-24[
0-24[
0-87[
0-248[
h
Fraction of genes
p=0.031
p=0.22
p=0.09
p=0.12
Log2ΔSILAC (WT/KO)
>100, n=270
50−100, n=282
25−49, n=449
1−24, n=1506
0, n=303
NXPM
i
p=0.15
p=0.014
p vs. CDS sites=0.11
Log2Δ(RPF/RNA abun.) (WT/KO)
CDS sites, n=88
median=−0.19
3'UTR sites, n=42
median=−0.032
0, n=732 median=−0.23

Supplementary Figure 6. Detailed integration of PAR-CLIP data and RNA/Ribo-seq. a, Same as in **Fig. 5a**, except mRNAs were binned based on the number of binding clusters. **b,** Same as in **Fig. 5a**, except mRNAs were binned based on the number NXPM in the 5' UTR. **c,** Same as in **a**, except FH-DHX36 wildtype PAR-CLIP data were used. **d,** Same as in **Fig. 5a**, except FH-DHX36 wildtype PAR-CLIP data were used. **e**, Same as in **Fig. 5b**, except FH-DHX36 wildtype PAR-CLIP data were used. **f,** Same as in **Fig. 5c**, except FH-DHX36 wildtype PAR-CLIP data were used. **g,** Same as **Fig. 5e** except the PURB mRNA binding site is depicted. **h,** Same as in **Fig. 5d**, except FH-DHX36 wildtype PAR-CLIP data were used. **i,** Cumulative distribution function comparing changes in DHX36 target mRNA abundance of DHX36 knockout cells (n=3) and parental HEK293 cells (n=3). DHX36-target mRNAs (determined by binding clusters) are separated by coding-sequence bound and 3' UTR bound.

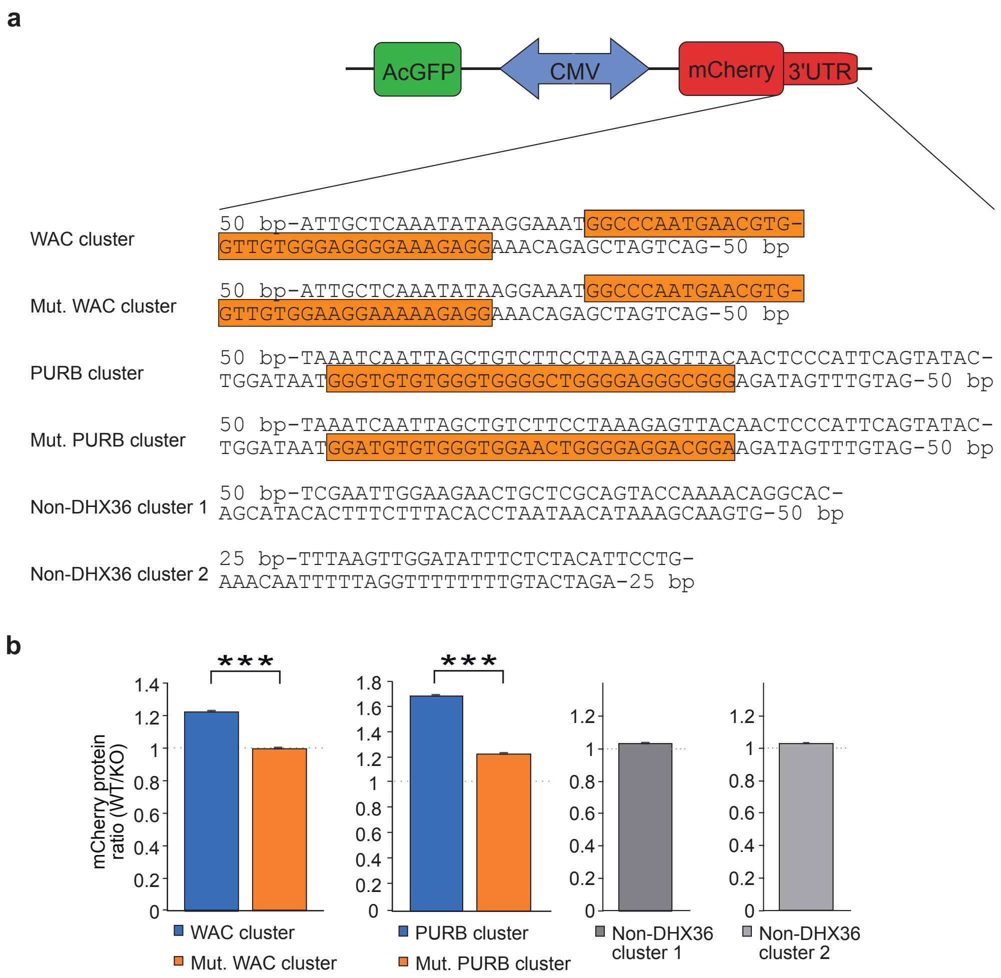

Supplementary Figure 7. Reporter assays for functional dissection of individual PAR-CLIP binding sites. a, Schematic representation of the reporter gene construct stable integrated in HEK293 wildtype and DHX36-KO cells. AcGFP and mCherry are co-expressed under the control of a bidirectional CMV promoter. Native WAC and PURB binding clusters (highlighted in orange) in their natural sequence context (+ 50 bp upstream and downstream) are indicated as well as mutated versions (G to A mutations in bold) and non-binding clusters of DHX36 **b,** Ratios of mCherry protein levels in WT and DHX36-KO cells expressed from the stably integrated reporter gene constructs determined by flow cytometry. Median fluorescence intensities (MFI) of three times 100,000 double positive cells were analyzed. Error bars represent standard deviations of three experiments. Significance was calculated using a Student´s T-Test (n=3) Significance levels: *$P<0.05$, **$P<0.01$, and ***$P<0.001$. Source data are provided as a Source Data file.

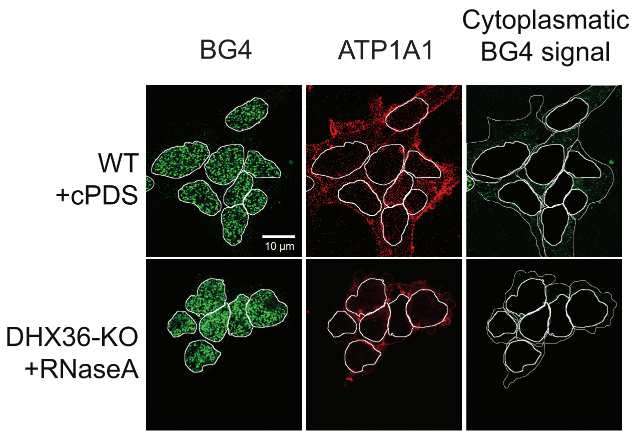

Supplementary Figure 8. Detailed microscopic images for the quantification in Fig. 6.

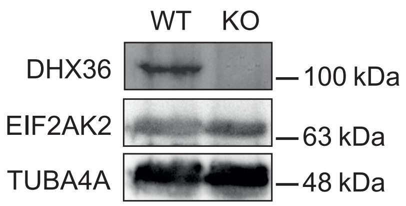

Supplementary Figure 9. DHX36 KO does not result in overall elevated PKR levels.

Western blot analysis of wildtype HEK293 cells (WT), DHX36-KO cells (KO) probed with antibodies directed against DHX36, PKR (EIF2AK2); and TUBA4A as loading control. Source data are provided as a Source Data file.

Supplementary Table 1

Oligonucleotides used in this study

Name	Sequence
Cloning of DHX36	
For DHX36	GGATCCGGATCCATGAGTTATGACTACCATCAG
Rev DHX36	CTCGAGCTCGAGTCAGCTGTAATATCCATCCTG
For DHX36-E335A	GTACTTGATGCAATCCATGAAAGAAATCTGCAGTCAG
Rev DHX36-E335A	CATGGATTGCATCAAGTACGATATGACTAACACTGGAC
For DHX36-Del14	GATCTCTTGATGTCACAAGTAATGTTTAAATCAGTTAACCAGACACAGGTGTT TAAAAGAACCCCTCCTGGTGTTCGGAAAATAGTAATTGC
Rev DHX36-Del14	GGGGTTCTTTTAAACACCTGTGTCTGGTTAACTGATTTAAACATTACTTGTGA CATCAAGAGATCATGTAAAGTGCTGATATTGTCCCAGCCTGG
Cloning of reporter constructs	
For GWC PURB	GGGGACAAGTTTGTACAAAAAAGCAGGCTTCATAGTAAGTGAATGAGATTATC AG
Rev GWC PURB	GGGGACCACTTTGTACAAGAAAGCTGGGTGCTGTATTTTCTTAAGGTAAATGT G
For GWC WAC	GGGGACAAGTTTGTACAAAAAAGCAGGCTTCAATGGACTTAAAAGTACTGCTG GATCGCTCAATGGACTTAAAAGTACTGCTG
Rev GWC WAC	GGGGACCACTTTGTACAAGAAAGCTGGGTGAACAATTTTTGTTTGTTTTAACA TG
For PURB mut RF	CAATTAGCTGTCTTCCTAAAGAGTTACAACTCCCATTCAGTATACTGGATAAT GAGTGTGTGGGTGAAGCTG
Rev PURB mut RF	CTTAAGGTAAATGTGTTTTTTTTTTTTTCTTTTTTCTTTTCTACAAACTATCT CTCGCTCTCCCCAGCTTC
For WAC mut RF	GACAGGCATGTGTGCTCAAAGTACATTGATTGCTCAAATATAAGGAAATGGCC CAATGAACGTGGTTG
Rev WAC mut RF	GTTTATTACAACAGATACAATTCACATCTGACTAGCTCTGTTTCCTCTTTCTT CTCTCACAAC
For non-target site 1	GGGGACAAGTTTGTACAAAAAAGCAGGCTTCAAATTTGTCATCAATTATGACT ACCC
Rev non-target site 1	GGGGACCACTTTGTACAAGAAAGCTGGGTGTTGGGATTAATTGCTTGATTAGC
For non-target site 2	GGGGACAAGTTTGTACAAAAAAGCAGGCTTCGTAATTATGGTGCACTTTTTCG
Rev non-target site 2	GGGGACCACTTTGTACAAGAAAGCTGGGTGTTTTGTGAAAACACTGCCTGC
CD oligonucleotides	
PAR-CLIP derived RRE	GGAGGAGGAGGAGG
mutated PAR-CLIP derived RRE 1	GAAGAAGGAGAAGAA
mutated PAR-CLIP derived RRE 2	GCAGCAGGAGCAGCA

TP-G4 (positive control)	GGGGGAGCTGGGGTAGATGGGAATGTGAGGG
WAC RRE	GGCCCAATGAACGTGGTTGTGGGAGGGGAAAGAGG
mutated WAC RRE	GGCCCAATGAACGTGGTTGTGAGAGAAGAAAGAGG
PURB RRE	GGGTGTGTGGGTGGGGCTGGGGAGGGCGGG
mutated PURB RRE	GAGTGTGTGGGTGAAGCTGGGGAGAGCGAG
NAA50 RRE	GGGGACTGCACAAGGATGTGAATACTGGGAGGTGG
mutated NAA50 RRE	GGAGACTGCACAAGGATGTGAATACTGAGAGGTGG
SLMO2 RRE	GGTGGGGAAGAACAAGCATAATGGTAGGGGGAGG
mutated SLMO2 RRE	GGTGGAGAAGAACAAGCATAATGGTAGAGAGAGG
MST	
DHX36 motif 5´ link Cy5	AAAAAGGAGGAGGAGGAGGA
DHX36 motif mut2 5´ link Cy5	AAAAAGCAGCAGGAGCAGCA
qPCR primer	
For RNU6 RT	GCTTCGGCAGCACATATACTAAAAT
Rev RNU6 RT	CGCTTCACGAATTTGCGTGTCAT
For NAA50 RT	TGGCACCTTACCGAAGGCTA
Rev NAA50 RT	TTGCCGACTCATTGCTGATCT
For PURB RT	GCCATCACCGTACCCTTCAAA
Rev PURB RT	CCCTCTGTCGTTCCTGGATTT
For WAC RT	GCCGGAGATCCTTCACCAC
Rev WAC RT	TTTGGCCTTACTGTGACCTGT
For SLMO2 RT	CCCAAACCCTATGAACCCAAG
Rev SLMO2 RT	TGTGCAACTTTCCAGAGGG
For mCherry RT	CCCGCCGACATCCCCGACTA
Rev mCherry RT	GGGTCACGGTCACCACGCC
