# Supplementary material for: DHX36 prevents the accumulation of translationally inactive mRNAs with G4-structures in untranslated regions
Source: Nat Commun. 2019 Jun 3;10:2421. doi: 10.1038/s41467-019-10432-5 (PMC6547686; doi:10.1038/s41467-019-10432-5)
Supplement: Supplementary file 3 — Description of Additional Supplementary Files [file 41467_2019_10432_MOESM3_ESM.pdf]

Description of Additional Supplementary Files

File Name: Supplementary Data 1
Description: Sequencing statistics of DHX36 PAR-CLIPs. DHX36_replicate_1.cluster (sheet 1); DHX36_replicate_2.cluster (sheet 2), DHX36_E335A_replicate_1.cluster (sheet 3); DHX36_E335A_replicate_2.cluster (sheet 4). Sequencing statistics, quality control, and mapping to RNA annotation categories are listed.

File Name: Supplementary Data 2
Description: In-depth PAR-CLIP analysis for individual genes. PAR-CLIP analysis (sheet 1). DHX36 binding clusters and NXPM are listed for each gene. DHX36E335A cluster 5mer Z-score (sheet 2). 5-mer scoring of the PAR-CLIP data obtained by using FH-DHX36-E335A. All possible 5-mers are listed as well as their target count and z-score. DHX36 WT cluster 5mer Z-score (sheet 3). 5-mer scoring of the PAR-CLIP data obtained by using FH-DHX36. All possible 5-mers are listed as well as their target count and z-score.

File Name: Supplementary Data 3
Description: Overlap of DHX36-PAR-CLIP data with published DHX36 targets. Published DHX36 target mRNAs and their occurrence in the PAR-CLIP data.

File Name: Supplementary Data 4
Description: RNA sequencing results. RNA sequencing results of WT and DHX36-KO cells (sheet 1). Transcripts are identified by their gene name. Transcript length and the lengths of their functional regions are listed. Results of RNA-seq experiments of wildtype and DHX36-KO cells are listed as well as the logarithmic change. RNA sequencing results of DHX36-rescue and DHX36-E335A rescue cells (sheet 2). Transcripts are identified by their gene name. Transcript length and the lengths of their functional regions are listed. Results of RNA-seq experiments of DHX36-KO wildtype rescue cells and DHX36-KO DHX35-E335A rescue cells are listed.

File Name: Supplementary Data 5
Description: Ribosome footprinting and SILAC results of WT and DHX36-KO cells. Ribosome footprinting results of WT and DHX36-KO cells (sheet 1). Transcripts are identified by their gene name. Transcript length and the lengths of their functional regions are listed. Results of Ribosome footprinting experiments of wildtype and DHX36-KO cells are listed as well as the logarithmic change. SILAC results of WT and DHX36-KO cells (sheet 2). Transcripts are identified by their gene name. Transcript length and the lengths of their functional regions are listed. Results of SILAC experiments of wildtype and DHX36-KO cells are listed as well as the logarithmic change.

File Name: Supplementary Data 6

Description: Chromatin-associated RNA and cPDS-treated RNA sequencing results of WT and DHX36-KO cells. Chromatin-associated RNA sequencing results of WT and DHX36-KO cells (sheet 1). Transcripts are identified by their gene name. Transcript length and the lengths of their functional regions are listed. Results of RNA-seq experiments of chromatin-associatd RNA of wildtype and DHX36-KO cells are listed as well as the logarithmic change. cPDS-treated RNA sequencing results of WT and DHX36-KO cells (sheet 2). Transcripts are identified by their gene name. Transcript length and the lengths of their functional regions are listed. Results of RNA-seq experiments of wildtype and DHX36-KO cells treated with cPDS are listed as well as the logarithmic change.
