# Supplementary material for: DHX36 prevents the accumulation of translationally inactive mRNAs with G4-structures in untranslated regions
Source: Nat Commun. 2019 Jun 3;10:2421. doi: 10.1038/s41467-019-10432-5 (PMC6547686; doi:10.1038/s41467-019-10432-5)
Supplement: Supplementary file 10 — Reporting Summary [file 41467_2019_10432_MOESM10_ESM.pdf]

nature research

Corresponding author(s): *Double-blind peer review submissions: write DBPR and your manuscript number here instead of author names.*

Reporting Summary

Nature Research wishes to improve the reproducibility of the work that we publish. This form provides structure for consistency and transparency in reporting. For further information on Nature Research policies, see Authors & Referees and the Editorial Policy Checklist.

Statistical parameters

When statistical analyses are reported, confirm that the following items are present in the relevant location (e.g. figure legend, table legend, main text, or Methods section).

n/a	Confirmed	
☐	☒	The exact sample size (*n*) for each experimental group/condition, given as a discrete number and unit of measurement
☐	☒	An indication of whether measurements were taken from distinct samples or whether the same sample was measured repeatedly
☐	☒	The statistical test(s) used AND whether they are one- or two-sided<br>*Only common tests should be described solely by name; describe more complex techniques in the Methods section.*
☒	☐	A description of all covariates tested
☒	☐	A description of any assumptions or corrections, such as tests of normality and adjustment for multiple comparisons
☒	☐	A full description of the statistics including central tendency (e.g. means) or other basic estimates (e.g. regression coefficient) AND variation (e.g. standard deviation) or associated estimates of uncertainty (e.g. confidence intervals)
☐	☒	For null hypothesis testing, the test statistic (e.g. *F*, *t*, *r*) with confidence intervals, effect sizes, degrees of freedom and *P* value noted<br>*Give P values as exact values whenever suitable.*
☒	☐	For Bayesian analysis, information on the choice of priors and Markov chain Monte Carlo settings
☒	☐	For hierarchical and complex designs, identification of the appropriate level for tests and full reporting of outcomes
☐	☒	Estimates of effect sizes (e.g. Cohen's *d*, Pearson's *r*), indicating how they were calculated
☐	☒	Clearly defined error bars<br>*State explicitly what error bars represent (e.g. SD, SE, CI)*

Our web collection on statistics for biologists may be useful.

Software and code

Policy information about availability of computer code

Data collection	Image Lab 5.2.1<br>Fractionator Software v8.04<br>Typhoon FLA 7000 Control Software<br>Illumina HiSeq3000<br>Spectra Manager Suite (Jasco)<br>CFX Manager 2.0 (Biorad)<br>StepOne software (Applied Biosystems)<br>Leica TCS SP5<br>BD FACSDIVA™ SOFTWARE
Data analysis	Image Lab 5.2.1<br>Coral Draw X8<br>Coral PhotoPaint X8<br>Adobe Illustrator CS5<br>Adobe Illustrator CS6<br>Microsoft Excel 2016<br>PARalyzer<br>MEME suite<br>IGV

R studio
CFX Manager 2.0 (Biorad)
Fiji
GraphPad Prism 6 FlowJo

For manuscripts utilizing custom algorithms or software that are central to the research but not yet described in published literature, software must be made available to editors/reviewers upon request. We strongly encourage code deposition in a community repository (e.g. GitHub). See the Nature Research guidelines for submitting code & software for further information.

Data

Policy information about availability of data

All manuscripts must include a data availability statement. This statement should provide the following information, where applicable:

- Accession codes, unique identifiers, or web links for publicly available datasets
- A list of figures that have associated raw data
- A description of any restrictions on data availability

PAR-CLIP, ribosome footprinting, and RNA-seq data have been deposited in the National Center for Biotechnology Information (NCBI) Sequence Read Archive under the accession number GSE105175.

Field-specific reporting

Please select the best fit for your research. If you are not sure, read the appropriate sections before making your selection.

[x] Life sciences [ ] Behavioural & social sciences [ ] Ecological, evolutionary & environmental sciences

For a reference copy of the document with all sections, see nature.com/authors/policies/ReportingSummary-flat.pdf

Life sciences study design

All studies must disclose on these points even when the disclosure is negative.

Sample size	All experiments were performed in biological triplicates, except for the PAR-CLIP experiments, which were collected as biological duplicates.
Data exclusions	No data were excluded from the analysis.
Replication	Experiments were performed in biological replicates.
Randomization	Not relevant to the study. No statistical analysis was performed that required allocation to different experimental groups.
Blinding	Not relevant.

Reporting for specific materials, systems and methods

Materials & experimental systems

n/a	Involved in the study	
[x]	[ ]	Unique biological materials
[ ]	[x]	Antibodies
[ ]	[x]	Eukaryotic cell lines
[x]	[ ]	Palaeontology
[x]	[ ]	Animals and other organisms
[x]	[ ]	Human research participants

Methods

n/a	Involved in the study	
[x]	[ ]	ChIP-seq
[ ]	[x]	Flow cytometry
[x]	[ ]	MRI-based neuroimaging

Antibodies

Antibodies used

Primary antibodies
DHX36 antibody: Mouse DDX36 antibody (B-6), Santa Cruz Biotechnology, ref# sc-377485
TUBA4A antibody: Monoclonal Anti-α-Tubulin antibody produced in mouse, clone B-5-1-2, Sigma-Aldrich, now Merck, ref# T5168-1000UL
HA antibody: Anti-HA.11 Epitope Tag Antibody, mouse, Biolegend, previously Covance, ref# MMS-101R
CANX antibody: Anti-Calnexin antibody, mouse, clone AF18, Abcam, ref# ab31290

HISTH2B antibody: Anti-Histone H2B antibody -ChIP grade, rabbit, Abcam, ref# ab1790
FMR1 antibody: Linder, B. et al. Tdrd3 is a novel stress granule-associated protein interacting with the Fragile-X syndrome protein FMRP. Hum Mol Genet 17, 3236-46 (2008).
RPL22 antibody: Ribosomal Protein L22 (52), mouse, Santa Cruz Biotechnology, ref# sc-136413
FLAG antibody: Monoclonal ANTI-FLAG® M2 antibody produced in mouse, clone M2, Sigma-Aldrich, now Merck, ref# F1804-200UG
BG4: Biffi, G., Tannahill, D., McCafferty, J. & Balasubramanian, S. Quantitative visualization of DNA G-quadruplex structures in human cells. Nat Chem 5, 182-6 (2013).
Biffi, G., Di Antonio, M., Tannahill, D. & Balasubramanian, S. Visualization and selective chemical targeting of RNA G-quadruplex structures in the cytoplasm of human cells. Nat Chem 6, 75-80 (2014).
ATP1A1 antibody: Anti-alpha 1 Sodium Potassium ATPase antibody [464.6], Abcam, ref# ab7671
ACTB antibody: Anti-ACTB antibody produced in rabbit, Sigma Aldrich, now Merck, ref# AV40173-50UG
FLAG-Antibody for immunofluorescence experiments: DYKDDDDK Tag Antibody (Binds to same epitope as Sigma's Anti-FLAG® M2 Antibody), Cell signaling, ref# 2368

Secondary antibodies
goat anti-mouse IgG-HRP (Cruz Marker), Santa Cruz Biotechnology, sc-2031
goat anti-rabbit IgG-HRP Cruz Marker™compatible, Santa Cruz Biotechnology, sc-2030
Goat anti-Mouse IgG (H+L) Cross-Adsorbed Secondary Antibody, Alexa Fluor 488, Life technologies, ref# A11001
Goat anti-Rabbit IgG (H+L) Cross-Adsorbed Secondary Antibody, Cyanine3 Life technologies, ref# A10520

Validation

DHX36 antibody: DDX36 (B-6) is recommended for detection of DDX36 of mouse, rat and human origin by Western Blotting [...].
TUBA4A antibody: Monoclonal Anti-α-Tubulin recognizes an epitope located in the C-terminal end of the a-tubulin isoform in a variety of organisms (e.g., human, sea urchin, Chlamydomonas). [...]. The product is useful in immunoblotting, [...].
HA antibody: The HA tag (hemagglutinin) is an amino acid sequence derived from the human influenza hemagglutinin surface glycoprotein, corresponding to amino acids 98-106. It is commonly used as a tag to facilitate detection, isolation, and purification of proteins. The full amino acid sequence is: YPYDVPDYA
CANX antibody: Reacts with: Mouse, Human. This antibody gave a positive signal in the following, in WB - HeLa whole cell lysate, A431, HEK293; in IF - HeLa cells; in IHC - human liver carcinoma; in FC - HeLa.
HISTH2B antibody: Synthetic peptide conjugated to KLH derived from within residues 100 to the C-terminus of Human Histone H2B. Suitable for: WB, IHC-P, ChIP, ICC/IF, IP
FMR1 antibody: Linder, B. et al. Tdrd3 is a novel stress granule-associated protein interacting with the Fragile-X syndrome protein FMRP. Hum Mol Genet 17, 3236-46 (2008).
RPL22 antibody: Ribosomal Protein L22 (52) is recommended for detection of Ribosomal Protein L22 of mouse, rat, human and canine origin by Western Blotting [...].
FLAG antibody: The ANTI-FLAG M2 mouse, affinity purified monoclonal antibody binds to fusion proteins containing a FLAG peptide sequence. The antibody recognizes the FLAG peptide sequence at the N-terminus, Met-N-terminus, C-terminus, and internal sites of the fusion protein. For highly sensitive and specific detection of FLAG fusion proteins by immunoblotting, immunoprecipitation, immunohistochemistry, immunofluorescence and immunocytochemistry.
BG4: Biffi, G., Tannahill, D., McCafferty, J. & Balasubramanian, S. Quantitative visualization of DNA G-quadruplex structures in human cells. Nat Chem 5, 182-6 (2013).
Biffi, G., Di Antonio, M., Tannahill, D. & Balasubramanian, S. Visualization and selective chemical targeting of RNA G-quadruplex structures in the cytoplasm of human cells. Nat Chem 6, 75-80 (2014).
ATP1A1 antibody: Reacts with: This antibody is specific for Na,K-ATPase alpha 1 subunit. Mouse, Rat, Sheep, Rabbit, Dog, Human, Pig, Xenopus laevis, Monkey
ACTB antibody: Anti-ACTB (anti-β-Actin) antibody recognizes an internal sequence of human β-actin.
FLAG-Antibody for immunofluorescence experiments: DYKDDDDK Tag Antibody detects exogenously expressed DYKDDDDK proteins in cells. The antibody recognizes the DYKDDDDK peptide (the same epitope recognized by Sigma's Anti-FLAG® antibodies) fused to either the amino- or carboxy-terminus of targeted proteins.

Eukaryotic cell lines

Policy information about cell lines

Cell line source(s)

Flp-In™ T-REx™ 293 Cell Line, Thermo Fisher Scientific, ref# R78007. All other cell lines used in this study are generated out of this paternal cell line

Authentication

Generated cell lines in this study were verified by Western blot, fluorescence activated cell sorting, and/or sequencing of genomic DNA

Mycoplasma contamination

All used cell lines are free of mycoplasma contamination

Commonly misidentified lines
(See ICLAC register)

Name any commonly misidentified cell lines used in the study and provide a rationale for their use.

Flow Cytometry

Plots

Confirm that:

☐ The axis labels state the marker and fluorochrome used (e.g. CD4-FITC).

☐ The axis scales are clearly visible. Include numbers along axes only for bottom left plot of group (a 'group' is an analysis of identical markers).

☐ All plots are contour plots with outliers or pseudocolor plots.

☐ A numerical value for number of cells or percentage (with statistics) is provided.

Methodology

Sample preparation	For mCherry expression, cell lines with stably integrated pcDNA5-FRT-GFP-mCherry-3pGW reporter constructs were induced with 500 ng ml-1 tetracycline (Merck) for 15 h. Cells were trpysinized, washed twice with PBS, and filtered through a 35 µm nylon net.
Instrument	LSRFortessa™ BD Biosciences
Software	BD FACSDIVA™ SOFTWARE and FlowJo
Cell population abundance	Over 90%
Gating strategy	FSC/SCC was gated to exclude cell duplets and to ensure a homogenous cell population

☐ Tick this box to confirm that a figure exemplifying the gating strategy is provided in the Supplementary Information.
